# Supplementary material for: Lived experiences of disabled individuals living in Bahir Dar City, North West Ethiopia, a phenomenological study
Source: PLoS One. 2023 May 19;18(5):e0284860. doi: 10.1371/journal.pone.0284860 (PMC10198501; doi:10.1371/journal.pone.0284860)
Supplement: S1 File — (DOCX) [file pone.0284860.s001.docx]

| **List of Codes** | **Definitions** |
| --- | --- |
| **Absence of elevator** | Unavailability of elevators in different institutions |
| **Absence of special needs professionals** | Unavailability of special need professionals in different service giving institutions and schools. |
| **Absence of support from government** | Non inclusiveness of disable community in the city administration plan and ignoring disable community |
| **Absence of family support** | Get difficulty to get family support in physical and economical aspect |
| **Absence of priority** | Not giving priority for disable community in different service giving organizations |
| **Accepting disability** | Thinking disability happening for the better to them |
| **Assisting Physically** | Local and city community help towards disable individuals in physical support |
| **Feeling of anxiety** | Feeling of persons with disability to be anxious in response to different physical, social and economic issue |
| **Boride** | Boride by other person saying |
| **Togetherness** | Celebration with community in different occasions |
| **Sucking lip** | Action of community reminding disability |
| **Contempt Disable person** | Contempt disable community for different economic and social role |
| **Community support in living environment** | Support of the community in locating directions and giving information including physical support |
| **Dependent on family** | Physical and economical dependent on family |
| **Dependent on others for movement** | Physical dependence for movement on strangers or friends |
| **Dependent on others for self-care** | Dependent on others for self-care including toileting and bathing |
| **Dependent on others for test** | Dependent on others for reading exams to them |
| **Disability seen as incapability** | Thinking disable persons as incompetent to do their own education or work by their own |
| **Discriminating bank system** | The act of asking eye witness for blind customers to get bank services(especially for withdrawing money) |
| **Divorcing** | Divorcing because of fearing disability |
| **Demoralizing** | Do not care about the moral of disable persons |
| **Feeding alone** | The community ignorance leads to drinking and eating in separate type of materials |
| **Economical problem to pay for rent** | Economical difficulty to live with wife and daughter |
| **Help less** | Unavailability of help in terms of physical economical and moral |
| **Expecting incompetent to marriage** | Experienced difficulty to get married from the community |
| **Families Ashamed of me** | False thinking and attitude towards disability and ashamed of by it |
| **Family support** | The support of disabled family members in physical mental social role and economical |
| **Fearing of community** | Fearing the community to seen with disability in public areas |
| **Fearing the future** | Worrying about future fate |
| **Fearing to touch disable person** | Community fear to get touched by disable person fearing communicability |
| **Giving up** | Loss of hope after incidence of unexpected conditions from individuals and service giving institutions |
| **Hate to disability** | Community perception towards disable community negatively and taking disability as a punishment |
| **Feeding by others hand** | Dependent on others for feeding |
| **Fear to approach** | Fearing the expectation of the community and stay apart from them |
| **Fear to marry disable** | By thinking disable person as incompetent unwilling to marry them |
| **Hurts feeling** | The expectation of negative thinking about disability |
| **Inadequate food** | Getting difficult the quality and quantity of food |
| **Keeping home alone** | The separation of disable persons from community locking inside home by family members |
| **Lack of inclusive ness** | The lack of participating disable persons in different public celebration and services |
| **Limited range of motion** | Inability to move by own self |
| **Limited in social activity** | Lack of freedom in participating different social activities |
| **Lack of money to learn** | Unable to pay school fee |
| **Listening FM radio** | Listening FM to forget bad feelings and emotions |
| **Listening music** | Listening music to forget anxiety |
| **Listening religious song** | Listening religious song to reassure and calm own self |
| **Little profit** | After work the amount of money from it to gain is small |
| **Looks unequal with well person** | The community drawing of disable persons |
| **Locking inside “gota’’** | Locked the disable individuals at home inside traditional store for harvest |
| **Misunderstanding disability situation** | Some community members draw disability in wrong sight |
| **My mother left me** | The occasion of left by mother alone while 2 years old disable boy |
| **Not able to get work** | Not getting work because of deafness |
| **Not satisfying in institutional service** | Not satisfied in institutional service |
| **Paid little** | The imbalance of work and payment |
| **Parents fear of community** | Parents fear to the community about their disable child to seen in the community |
| **Participating in associations** | The disable persons participation in disability association meetings |
| **Poor work chance** | Poor expectation about disability to work |
| **poor enforcement of law** | The law doesn’t support disable community as the law |
| **Difficulty for rent** | Not volunteer to rent disable person |
| **Road problems** | Poor infrastructure for disability |
| **People afraid of feeding together** | People afraid of feeding together fearing communicability of disability |
| **Partner support** | Support of economy and physical |
| **Prohibited to work on the street** | Prohibited to work on the street leads to get little income |
| **Punishment from God** | Drawing disability as punishment from GOD |
| **Self-reassurance by others sever disability** | Self-reassurance by looking others sever disability related to them |
| **Prohibiting from social activities** | Community undermining disable persons and not letting them to participate in different social role |
| **Sign language translator problem** | Communication barrier for deaf persons in different institutions secondary to absence of sign language translator |
| **Stressful condition for learning** | Social economical and physical condition that makes to worry on it rather attending class free of stress |
| **Tired to do job** | The physical condition of disable persons not to able perform labor works as others |
| **Unsafe class rooms to going up** | The physical barrio to attend class rooms steepness and being on the upper part of buildings |
| **Using unsafe water** | The cost of living increments forced disable persons to use lake waters for self-cleansing purpose |
| **Work makes not to be sad** | Passing time with work make disable community not to be in bad mood and make them happy |
| **working place problem** | Different type of prohibition and non-availability of work place including uncomfortable work place |
| **watching movie** | Watching movie to forget bad feelings |
