# Supplementary material for: Lived experiences of disabled individuals living in Bahir Dar City, North West Ethiopia, a phenomenological study
Source: PLoS One. 2023 May 19;18(5):e0284860. doi: 10.1371/journal.pone.0284860 (PMC10198501; doi:10.1371/journal.pone.0284860)
Supplement: S2 File — (DOCX) [file pone.0284860.s002.docx]

Interview guide English Version

**Section one. Socio Demographic Questions**

1. How old are you?
2. Sex…….
3. Are you married?
4. Which religion you follow?
5. What is your level of education?
6. What type of disability you have?
7. How many years of life you live with disability?

**Section two:** Interview guide

1. What is disability to you?

- How the community does look disability in general?
- What does it have to do with spiritual belief?
- Daily activities (office services banks related to law?)

1. How was your disability happened?

- How long you lived with disability?
- How the communities think your cause of disability?

1. What is your source of income?

- Is it suitable related to your disability?
- Is there any difficulty to you to get Job?
- Is there any discrimination related to your disability to get job?

1. Because of your disability have you ever face discrimination tell us in detail?

- For marriage
- How you cope when stress full conditions happened to you

1. Tell us about your lifestyle?

- How do you participate in social activities?

1. How do you feel living with disability?
2. What support has you from family, partner, and friends or community?

- What opportunities do you have?

1. Is there anything we missed that you would like to tell us about living with disability?

**Thank you for participating in this study.**
