# Supplementary material for: Lived experiences of disabled individuals living in Bahir Dar City, North West Ethiopia, a phenomenological study
Source: PLoS One. 2023 May 19;18(5):e0284860. doi: 10.1371/journal.pone.0284860 (PMC10198501; doi:10.1371/journal.pone.0284860)
Supplement: Attachment — Submitted filename: Reviwers comements to the auther_PONE-D-23-03819.docx [file pone.0284860.s003.docx]

Reviewers comment

Date 6 March 2023

Title: Lived Experiences of Disabled Individuals Living in Bahir Dar City, North
West Ethiopia, A Phenomenological study

Manuscript number: PONE-D-23-03819

Summary of the research and the overall impression

Title: A very impressive area of research that emphasized a special population group(disabled people) is a sound and good research area. A manuscript is also structured and written very well. Also, the article is presented in an intelligible fashion; however, the English language has to be edited further by a native English speaker.

Abstract: Exceeds 300-word counts and it may be beyond the journal requirement. No recommendations were made in the conclusion section of the abstract. I advise the author to include pertinent recommendations in the abstract section.

2. Discussion of specific areas for improvement

Minors issues

Introduction: The last paragraph says “Even the research done here in Bahir Dar recommends further study so that this study aimed to gain an understanding of the essence of the experience of living with disability”. Does it mean other research previously conducted on the same topic at Bahir Dar? If, what additional finding can the current study address to the scholarly community??

In the introduction or background section, the author needs to include a paragraph that shows problems related to the study phenomenon at Bahirdar, then he has to indicate the justification or aim for this study.

Method section

In the sentence “Then we got o all sub-city social affairs offices….” You have to remove “o” ? from the Population and sample heading in the fourth paragraph?

In the method section under scientific rigor, the author missed ensuring the reflexivity of the study. Reflexivity is essential in qualitative research because this field is heavily dependent upon the information that participants provide. Since questionnaires, discussions, and interviews are all led by researchers, the information gathered during qualitative studies may be influenced by underlying beliefs. So, make sure you’re actively reflecting on yourself and understand how your personal experiences impact every decision you make in your research. Do you have to explain how you maintained it?

**In analysis section**

Colaizzi's phenomenological analysis can be used reliably to understand people's experiences in descriptive phenomenology. Why did you use thematic over Colaizzi's phenomenological analysis for this research?

**Result section**

Sociodemographic table, I advise the author to remove the source of income column, it may expose the participants. It is better to anonymize the participant for ethical reasons?

**Themes and subthemes**

Under this section, tables of theme and sub-theme including a list of codes under each theme or codebook have to be uploaded as supporting information.

Discussion

This section could be started after indicating the brief objectives of the research followed by indicating the main finding of the study.

Under the conclusion section, this research has no recommendations. I advise the author needs to include the recommendation.

Generally, this paper is written very well and structured scientifically sound. It will add good pieces of evidence to the body of already available studies.
